# Fusion Proteins of NKG2D/NKG2DL in Cancer Immunotherapy

**DOI:** 10.3390/ijms19010177

**Published:** 2018-01-07

**Authors:** Hui Ding, Xi Yang, Yanzhang Wei

**Affiliations:** Department of Biological Sciences, Clemson University, 190 Collings Street, Clemson, SC 29634, USA; ding4@clemson.edu (H.D.); xiy@clemson.edu (X.Y.)

**Keywords:** fusion protein, NKG2D, NKG2D ligands, NKG2D CARs, cancer immunotherapy

## Abstract

NKG2D (natural killer group 2, member D) is an important activating receptor in natural killer (NK) cells and some T cells. NKG2D ligands (NKG2DLs) are specifically expressed on most tumor cells. The engagement of these ligands on tumor cells to NKG2D on NK cells will induce cell-mediated cytotoxicity and have target cells destroyed. This gives NKG2D/NKG2DLs great potential in cancer therapeutic application. The creation of NKG2D/NKG2DL-based multi-functional fusion proteins is becoming one of the most promising strategies in immunotherapy for cancer. Antibodies, cytokines, and death receptors have been fused with NKG2D or its ligands to produce many powerful fusion proteins, including NKG2D-based chimeric antigen receptors (CARs). In this article, we review the recent developments of the fusion proteins with NKG2D/NKG2DL ligands in cancer immunotherapy.

## 1. Introduction

Natural killer (NK) cells activating receptor NKG2D (natural killer group 2, member D) and NKG2D ligands (NKG2DLs) play critical roles in cancer immune surveillance, which is one of the most significant findings in the fight against cancer in recent decades [1,2,3,4]. NKG2D, encoded by the gene KLRK1, was first found on the surface of NK cells as an immunosurveillance receptor. It was also found in CD8+ (cluster of differentiation) cytotoxic T cells, some NKT cells, some γδ T cells, and a small subset of CD4+ cytotoxic T cells [4,5,6,7]. NKG2D is a type II transmembrane protein. In humans, two NKG2D proteins recruit four hematopoietic cell signal transducer (DAP10) adaptor proteins to form a hexameric receptor complex. NKG2D associates with DAP10 adaptor proteins by ionic interaction in their transmembrane segments. The two NKG2D ectodomains in the hexameric receptor complex serve as ligand binders. When binding to ligands, this receptor complex triggers the activation of NK cells through the PI3K (phosphatidylinositol-3 kinase) and Grb2-Vav1 (growth factor receptor-bound protein 2, vav guanine nucleotide exchange factor 1) signaling pathways to promote Ca^2+^ influx, actin cytoskeleton reorganization, and microtubule polarization. As a result, the contents in the granules of NK cells are released to induce the apoptosis of target cells.

Although NKG2D is a quite conserved receptor, NKG2DLs are very diverse. They are structurally similar to major histocompatibility complex (MHC) class I proteins (MIC family), which are highly polymorphic [8]. MICA [4] and MICB [2] are the most extensively studied NKG2DLs in humans. Other NKG2DLs include UL16 binding proteins (ULBPs) [9] in human and mouse, murine UL16-binding protein-like transcript 1 (MULT1) [10], retinoic acid early transcript 1 (Rae1) [11,12] and histocompatibility antigen 60 (H60) [13] in mouse. NKG2DLs are rarely expressed in healthy cells. They are induced when the cell is under the stress of virus infection or malignant transformation, and therefore are called “induced-self” ligands [14]. Cells expressing these ligands will be determinedly detected and eliminated by NK cells. The diversity and the inductivity of NKG2DLs provide NK cells with an effective mechanism in immunosurveillance. However, advanced tumor cells tend to down-regulate or shed off NKG2DLs to escape immune elimination [15].

The combination of the specificity of NKG2DLs on stressed cells, the ability of the NKG2D/NKG2DLs pathway to active NK cells, and the powerful cytotoxicity of NK cells to target cells provides a great application potential of NKG2D/NKG2DLs in cancer immunotherapy. A great number of studies applying NKG2D/NKG2DLs in cancer immunotherapy have been conducted. Firstly, direct up-regulation of NKG2DLs in cancer cells is a straightforward strategy. NKG2DLs have been ectopically expressed in many tumor cell lines, which suppressed the establishment of tumors in vivo [11,16]. Many drugs, such as all-trans retinoic acid (ATRA) [17,18], trichostatin A [17], vitamin D3 [17], and some histone deacetylase inhibitors [19,20] were reported to up-regulate NKG2DLs and could be potential treatments for cancers. Some viral proteins (e.g., E1A in adenovirus), when expressed in tumor cells could up-regulate NKG2DLs and reduce the tumorigenicity of the tumor cells [21,22]. Some chemotherapeutic agents or radiotherapies, as a side-effect, could also induce up-regulation of NKG2DLs by causing DNA damage [23]. Secondly, the expression of NKG2D in NK cells can also be up-regulated as a possible treatment for cancer. The gamma-chain containing cytokines, such as interleukin (IL)-2 [24], IL-7 [25], IL-12 [26], and IL-15 [27], were reported to increase NKG2D expression in human and mouse NK and CD8+ T cells. An IL-15 superagonist mutant (N72D) alone or associated with a dimeric IL-15 receptor α/Fc fusion protein was found to significantly up-regulate NKG2D expression in NK cells and CD8+ T cells [28,29]. Other cytokines, like IL-21 [30], interferon (IFN)-γ [31], and transforming growth factor (TGF)-β [32] were reported to decrease NKG2D expression. Lastly, the association between the polymorphism of NKG2DLs and its genetic predisposition to different cancer types [33] can also be investigated in cancer prediction and therapy.

## 2. Strategies of Fusion Proteins

**Figure 1:**
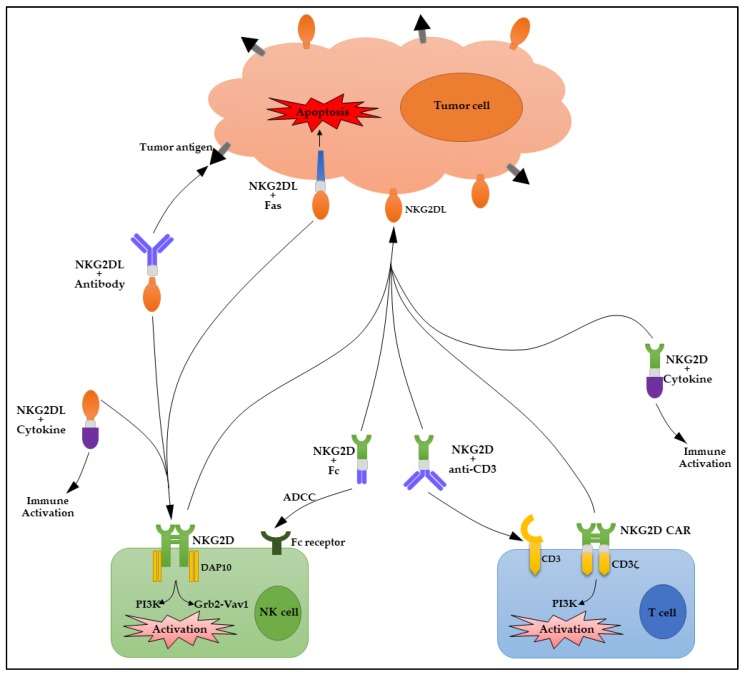
Schematic diagram of fusion proteins of NKG2D (natural killer group 2, member D)/NKG2D ligands (NKG2DLs) in cancer immunotherapy. Fc, fragment crystallizable region of an antibody; ADCC, antibody-dependent cell-mediated cytotoxicity; CD3, cluster of differentiation 3; DAP10, hematopoietic cell signal transducer; PI3K, phosphatidylinositol-3 kinase; Grb2, growth factor receptor-bound protein 2; Vav1, vav guanine nucleotide exchange factor 1; CAR, chimeric antigen receptors.

The generation of multi-functional fusion proteins consisting of NKG2D/NKG2DLs has become a very active research area in developing effective cancer immunotherapies using various strategies (Figure 1). NKG2D/NKG2DLs fusion proteins involving monoclonal antibodies, cytokines, death receptors, and chimeric antigen receptors are all discussed in this review (Table 1).

### 2.1. NKG2DL + Antibody

There have been more than a dozen monoclonal antibodies (mAbs) approved by the US Food and Drug Administration (FDA) to treat certain cancers [34]. They can be just naked mAbs that label cancer cells to the immune system, boost immune response against the cancer cells (e.g., antibody-dependent cell-mediated cytotoxicity (ADCC)), or block the antigens that stimulate cancer cell growth. The antibodies can also be conjugated with chemotherapy drugs or radioactive particles to enhance the delivery accuracy to cancer cells. Two different antibodies can also be combined together to bridge cancer cells and immune cells, facilitating immune responses. Fusing a NKG2DL to a tumor-targeting antibody will create a bispecific fusion protein to bridge NK cells and tumor cells, which will redirect and trigger NK cells against the tumor cells.

NKG2DLs usually used in this strategy include H60 [35,36] and Rae1β [35,37] in mouse, and MICA [38,39,40,41] and ULBP2 [38,42,43,44] in human. Since this kind of fusion proteins is always secreted, only the extracellular domain of the NKG2DLs are used. Many antibodies were used against various tumor antigens, such as TNT-3 [35,36] against universal nuclear antigen [45], BB4 [42] against CD138 (syndecan-1), 7D8 [38] against CD20, rG7S [39] against CD24, and antibodies against human epidermal growth factor receptor 2 (HER2) [37], prostate carcinoma membrane antigen (PSMA) [43] and carcinoembryonic antigen (CEA) [44]. As angiogenesis plays an important role in the progression of solid cancer, antibodies against vascular endothelial growth factor receptor (VEGFR) [39,46] were also used in this strategy.

The NKG2DL gene could be fused with the antibody heavy chain gene and co-transfected with the light chain gene to produce a full antibody fused with NKG2DL [35]. The NKG2DL gene, heavy chain gene, and light chain gene could all be linked together by some linker sequences to become a single fusion gene. Or only a fragment of immunoglobulin—such as the single-chain variable fragment (scFv) or crystallizable fragment (Fc)—was used to fuse with the NKG2DL [38,39,42]. The Fc portion in the fusion protein can bind to the Fc receptors (CD16) on NK cells, transmitting activating signals into NK cells. After activating, NK cells can lyse the target cells and secrete cytokines to induce adaptive responses [47]. Germain et al. [40] created a fusion protein with the extracellular domain of MICA and Fc region, and then chemically conjugated it with the Fab fragments of three monoclonal antibodies against tumor-associated antigens: anti-CEA against carcinoembryonic antigen [48], trastuzumab against HER2 [49], and rituximab against CD20 [50].

The NKG2DL-antibody fusion proteins are all soluble. *E. coli*, yeast, and mammalian cells (e.g., 293T) are used to produce them. They are then purified, concentrated, and used to treat the antigen-positive tumor cells. These fusion proteins showed great binding specificity to the target cells due to the antibody activity. Meanwhile, their NKG2DL portion triggered the cytotoxicity of NK cells against the tumor cells. The Fc portion of the antibody also induced ADCC [38]. Anti-VEGFR-NKG2DL fusion protein demonstrated superior anti-tumor efficacy in a mouse model bearing human breast tumor [39], and induced antiangiogenic activities in vitro [46].

### 2.2. NKG2DL + Cytokine

Some activating cytokines have essential roles in maintaining cytotoxic lymphocytes’ function and increasing their sensitivity and capability to lyse tumor cells [51], and consequently are used in cancer therapy. For example, IL-2 has been used in the treatment of several malignancies for 30 years [52]. IL-12 is even more efficient than IL-2 in activation of NK cells [53], and also has a potent anti-tumor activity through augmentation of the cytotoxic activity of NK cells, activation of CD8+ T cells, and the inhibition of angiogenesis [54,55]. In order to enhance the functions of these cytokines, they are fused with NKG2DLs which can guide the cytokines to the NKG2D-expressing NK cells or CD8+ T cells, subsequently activating them through both the cytokine/receptor pathway and the NKG2D/NKG2DL pathway.

Tietje et al. [56] in 2014 fused mouse IL-12 and the mouse MULT1E (extracellular domain of MULT1) to create a bi-functional protein. MULT1E binds to NKG2D receptor to activate NK cells, and simultaneously IL-12 engages the IL-12 receptors on NK cells to enhance the cytotoxicity of the killer cells against the tumor cells. The gene of this fusion protein was transfected into tumor cells, and a tumor cell line that stably secreted the fusion protein was generated. When co-culturing this tumor cell line with NK cells, IFN-γ was over-produced and NK cells were highly activated. When intravenously injecting the tumor cell line expressing this fusion protein into a mouse model, the tumor genesis and growth were suppressed compared to injecting the wild type tumor cells. After demonstrating the validity of this strategy in mouse, Tietje et al. [57] created a human version of fusion protein by fusing human IL-12 with the extracellular domain of human MICA. This fusion protein was more effective than either IL-12 or MICA alone in priming NK cells, and was able to increase the proliferation of human peripheral blood mononuclear cells (PBMCs) and their production of IFN-γ.

IL-2 was also applied in creating fusion proteins recently. In order to improve the safety of using IL-2, a mutant form of IL-2 was used which had a poor affinity to the IL-2 receptor. It was fused with a special NKG2DL, orthopoxvirus major histocompatibility complex class I-like protein (OMCP) from cowpox virus [58]. The ligand OMCP had a greater affinity with NKG2D than the other NKG2DLs, and could bind to both human and murine NKG2D [59]. It helped the mutant form of IL-2 to selectively activate the IL-2 signaling pathway only on NKG2D-bearing cells (e.g., NK cells). This fusion protein significantly reduced the broad toxicity and vascular complications associated with the wild type IL-2, and provided a superior NK cell-mediated cancer immunotherapy.

### 2.3. NKG2DL + Fas

One hallmark of cancer is evading apoptosis (programmed cell death). Cancer cells can resist apoptosis by secreting antagonistic decoy receptors [60], expressing anti-apoptotic molecules (such as BCL2 family members [61]), down-regulating and mutating pro-apoptotic genes (such as BAX, APAF1, or Fas [62,63,64]). Fas (CD95) is a death receptor that transduces apoptotic signals into the cells by forming death-inducing signaling complex (DISC) [65]. Its ligand (FasL) is produced by activated T cells and NK cells [60]. Fas/FasL interaction plays a critical role in the immune progression of cancer [66]. Applying Fas to NKG2DLs fusion protein can graft its apoptosis-inducing function on the fusion protein to make it more powerful.

Kotturi et al. [67] designed and evaluated a fusion protein—MULT1E-FasTI—which fused the extracellular domain of MULT1 and the transmembrane and intracellular domains of Fas. This fusion gene was transfected into a mouse tumor cell line. The expressed protein MULT1E-FasTI was anchored on the cell surface. When treated with recombinant protein NKG2D-Fc, apoptosis of the tumor cells was induced. NK cells were also activated in the co-culture with these tumor cells. Tumor cells expressing MULT1E-FasTI proliferated significantly more slowly compared to tumor cells without the fusion protein in mouse. Kotturi et al. [68] packaged this fusion gene in an adenoviral delivery vector and directly injected the viral particles into the tumors in mice. This treatment induced the apoptosis of the tumor cells in vivo and significantly delayed the tumor growth.

### 2.4. NKG2D + Antibody

Antibodies can also be fused with NKG2D. These fusion proteins use the NKG2D portion to target tumor cells and use the Fc portion of the antibody to induce ADCC against tumor cells. Salih’s group [69] generated a fusion protein of this kind, consisting of the human NKG2D extracellular domain and the human IgG1 Fc portion. They modified the Fc with S239D/I332E to greatly increase its affinity with the Fc receptors on NK cells. The fusion protein highly enhanced the ADCC, degranulation, and IFN-γ production of NK cells in response to breast cancer cells, despite the fact that the NKG2D portion may block some of the NKG2DLs on the cancer cells and decrease their exposure to NK cells. This fusion protein represents a more attractive potential in the immunotherapy to HER2/neu-low or -negative breast cancer compared to the anti-breast cancer antibody trastuzumab, because it did not target the HER2 antigens but the more widely expressed NKG2DLs. Salih’s group [70] also used similar NKG2D-Fc fusion proteins with modifications in the Fc part to mediate ADCC of NK cells against leukemia. Wu et al. [71] generated two versions (mouse and human) of Dap10-Fc-NKG2D by fusing Dap10 signal peptide, IgG1 Fc (Hinge-CH2-CH3), and NKG2D extracellular domain. They used iron oxide nanoparticles (IONPs) [72] as vehicles to deliver their fusion proteins to multiple NKG2DL-positive tumor cells in vitro.

Sentman’s group [73] used another strategy to create a fusion protein anti-CD3 scFv-NKG2D, in which anti-CD3 single-chain variable fragment was used to target CD3 on T cells. This fusion protein engaged T cells to tumor cells, stimulating T cells to produce IFN-γ and induce cytotoxicity against NKG2DL-positive tumor cells in vitro. A significantly improved anti-tumor activity and survival promotion was observed in vivo.

### 2.5. NKG2D + Cytokine

Many cytokines, like IL-2, IL-15 and IL-21 were chosen to fuse with NKG2D because of their ability to promote immune cell functions and induce anti-tumor responses. The fusion protein NKG2D-Fc-IL-2 was first designed to use NKG2D to deliver IL-2 to the tumors, in order to achieve a local high-dose in the microenvironment and avoid the severe side effects of systemic administration of IL-2 [74]. Its delivery of IL-2 to the tumor location in vivo was significant, and it enhanced antigen-specific T cell immune response and controlled tumor growth.

IL-15 plays an important role in the development, activation, and persistence of NK cells and CD8+ T cells. Gong’s group [75] generated the fusion protein dsNKG2D-IL-15 consisting of two identical extracellular domains of human NKG2D and human IL-15. This fusion protein targeted the NKG2DL-positive tumor cells through its NKG2D domains and presented IL-15 to NK or CD8+ T cells to promote their activation and cytotoxicity to tumor cells. They found that it significantly retarded the growth of transplanted colon cancers [75] and melanoma [76] in mouse tumor models. Its efficiency in suppressing cancer growth was better than IL-15 cytokine therapy alone. They also delivered their fusion gene into tumor cells by loading the DNA with chitosan nanoparticles [77]. Tumor cells uptaking the nanoparticles with the fusion gene stimulated NK cells and CD8+ T cells in vitro. Intramuscular injection of the nanoparticles with the fusion gene also suppressed tumor growth and prolonged the survival of tumor-bearing mice.

IL-21 is mainly produced by activated T cells and modulates the functions of T, B, and NK cells. IL-21/IL-21R signal pathway regulates both innate and adaptive immune systems, and is significant in inflammatory responses, the development of autoimmune diseases and inflammatory disorders, anti-tumor and antiviral response [78,79]. Tan et al. [80] fused two copies of NKG2D extracellular domain cDNA with IL-21 gene in a vector, and used chitosan nanoparticles to deliver this fusion gene vector into tumor cells. Tumor cells treated with the nanoparticles secreted the fusion protein, which activated NK and CD8+ cells to lyse tumor cell in co-culture. Intramuscularly injecting these NKG2D-IL-21 nanoparticles increased IL-21 levels in serum and promoted the activation of many lymphocytes (including NK cells). After intravenous injection, these NKG2D-IL-21 nanoparticles labeled with fluorescein were detected accumulating and remaining in the tumor tissues of mouse. Tumor growth was sharply retarded, and the life span of tumor-bearing mice was prolonged after injection.

### 2.6. NKG2D CARs

Chimeric antigen receptors (CARs) are engineered receptors that graft an extracellular antigen-recognition domain onto an intracellular signaling domain in immune effector cells (usually T cells) [81]. The extracellular antigen-recognition domain usually is a single-chain variable fragment derived from monoclonal antibodies. The intracellular signaling domain in the first-generation CARs is typically the CD3ζ signaling chain of T cell receptors (TCRs) [82]. The T cells expressing CARs become antigen-specific cells that are able to recognize specific antigen-expressing cells and subsequently stimulate T cell proliferation, cytokines release, and cytotoxicity to kill the target cells. The second-generation CARs added an additional co-stimulatory signaling domain (e.g., CD28 or 4-1BB) to the first-generation CARs, which significantly enhanced the persistence and proliferation of CAR T cells [83]. The second-generation CD19-specific CAR T cells have demonstrated clinical efficacy in the treatment of B-cell acute lymphoblastic leukemia [84]. The third-generation CARs added two additional co-stimulatory signaling domains to the first-generation CARs, which were even better than the second generation in the anti-tumor efficacy [85]. CARs can basically graft any antigen-recognition capability to T cells, but so far the clinical trials of CARs therapy have been only in patients with blood cancers.

NKG2D as a receptor targeting tumor cells can replace the scFv portion in the typical CARs. In 2005, Sentman’s group [86] first reported this NKG2D CAR, which fused murine CD3ζ chain cytoplasmic region and the full-length murine NKG2D. They used retroviruses to transduct this fusion gene into mouse primary T cells. These T cells responded to tumor cells expressing NKG2DLs in co-culture, produced a large amount of T-helper 1 (Th1) cytokines and proinflammatory chemokines, and induced the lysis of tumor cells bearing NKG2DLs. T cells with this NKG2D CAR inhibited the tumor growth in vivo and showed resistance to new tumor cell challenge. Sentman’s group also created a human version of this fusion gene and applied it on primary human T cells [87]. Similar anti-tumor responses were also observed. The same strategy was also used effectively against not only multiple myeloma, ovarian carcinoma, and lymphoma in vitro and in vivo [88,89,90,91], but also tumor vasculature expressing NKG2DLs [92] and heterogeneous tumors consisting of NKG2DL-expressing and ligand-deficient tumor cells [93]. The group went further and investigated the molecular mechanisms of their NKG2D CARs in cellular cytotoxicity, cytokine production, and the host immune response [88,94,95,96]. In one of their recent studies, they found that high-dose injection of NKG2D CAR T cells resulted in cytokine release syndrome-like responses in mice, which were similar to those seen in some patients given CAR T cells therapy [97].

Lehner et al. [98] and Song et al. [99], respectively, grafted the extracellular domain of NKG2D on two signaling platforms CD28-CD3ζ or 4-1BB-CD3ζ to create new NKG2D CARs. CD28 and 4-1BB (CD137) were added to improve the proliferation and persistence of CAR T cells. CD28 increased IL-2-independent proliferation, and 4-1BB served as a TNF receptor. Upon stimulation with tumor cells, both primary CD4+ T cells and CD8+ T cells expressing these two CARs were activated and demonstrated high cytotoxicity in vitro. Chang et al. [100] added DAP10 to create the fusion protein NKG2D-DAP10-CD3ζ and applied it to NK cells rather than T cells. It markedly activated NK cells, induced greater cytotoxicity against a wide spectrum of tumor subtypes, and produced more cytokine types than mock-transduced NK cells.

VanSeggelen et al. [101] constructed a fusion protein with full-length murine NKG2D and cytoplasmic murine CD3ζ, and engineered it into murine T cells. They found that by adding an adaptor protein (DAP10) to this fusion protein, the protein expression of NKG2D-CAR would be increased 7–10 fold. They also constructed a second-generation CAR that fused the extracellular domain of NKG2D with the CD8 hinge region, the CD28 transmembrane and cytoplasmic domains, and the CD3ζ cytoplasmic domain. All these NKG2D-CARs showed strain-specific differences in surface expression and in vitro functionality. After adoptive transfer of NKG2D-CAR T cells into model mice, they observed dramatic toxicity symptoms—including acute cytokine storm and mortality—in both breast tumor-bearing mice and tumor-free mice. The NKG2D-CAR with DAP10 showed most severe toxicities, which paralleled its highest NKG2D-CAR expression.

## 3. Discussion

Because the advanced tumor cells can intentionally reduce the expression of NKG2DLs or even shed them off to escape immunosurveillance, the NK cells’ efficiency of detecting the ligands and activating cytotoxicity to kill tumor cells becomes questionable. To overcome this problem, artificially increasing the NKG2DLs on tumor cells or in the tumor microenvironment would be a straightforward thinking. Fusing the ligands with tumor-associated antigen-specific antibodies would help the NKG2DLs to access and label tumor cells. However, this strictly depends on the expression of the antigens on the tumor cells. For instance, high expression of HER2 occurs only in 25–30% of breast cancer patients [69]. Tumor cells’ heterogeneity and evading down-regulation of the antigens also need to be considered. Fusing NKG2DLs to cytokines promotes the delivery of cytokines to NK cells and increases cytokines’ efficiency. These fusion proteins target not tumors but NK cells. The activation from both NKG2DLs and cytokines may cause overreaction of NK cells, and then systemically hyper immune responses, not to mention that the activating cytokines themselves have many side effects. For instance, high dose IL-2 can activate resting cytotoxic lymphocytes, causing wide toxicities [58]. Although the fusion protein MICA-Fas combining NK cell activation and apoptosis induction was powerful against tumor, a significant problem is the specific delivery of the fusion gene to tumor cells. Direct injection of viral vector carrying the fusion gene into the tumor is one approach, but the safety must be considered.

All the fusion proteins with NKG2D have so far used the NKG2D portion to target tumor cells. Their targeting to the tumor cells is very dependent on the expression of NKG2DLs on tumor cells. However, as we mentioned before, there are plenty of problems with NKG2DLs on tumor cells, such as expression down-regulation, shedding, and variance in tumor types and stages [102,103,104,105]. Shedding will release soluble ligands such as MICA. Binding to soluble MICAs can markedly reduce the expression of NKG2D and severely impair the responsiveness of NK cells [106]. Multiple reports showed that NKG2DLs are expressed in various healthy, noncancerous tissues [101]. Some drug treatments and anti-cancer therapies (e.g., chemotherapy) could also induce their expression in noncancerous tissues. Therefore, many factors must be taken into consideration with caution prior to the clinical applications of these NKG2D fusion proteins to avoid unwanted severe toxicities. This is not only a low-efficiency or off-target problem, but also a safety problem. A strategy to avoid the NKG2DLs side effect would be to keep the transmembrane and intercellular domains and replace the extracellular domain of NKG2D with some other proteins to target the specific antigens or proteins on tumor cells (such as antibodies). This fusion protein will utilize the activation signaling pathway of NKG2D in NK cells, but is not limited to targeting NKG2DLs.

Most of the fusion proteins we discussed are soluble, such as NKG2DLs or NKG2D fused with antibodies or cytokines. They are easy to produce and manipulate on treatment. However, in order to express fusion proteins on the tumor cells, accurate delivery of the fusion genes to the tumor cells is one of the most critical challenges. Viral (including lentiviral [107] and adenoviral [108]) delivery systems and nanoparticles [109] are available delivery methods so far. The chitosan nanoparticle is a popularly used vehicle and shows great potential in cancer therapy, because of its capability of tumor targeting and releasing drugs or genes automatically in tumor tissues [80]. However, this is still far from perfect. If not tightly regulated or controlled, the ectopic expression of these NKG2DL fusion proteins may trigger overactivation of the immune system, leading to autoimmune responses or other serious problems.

CAR therapy for cancer is one of the most promising strategies in cancer immunotherapy. It has been restricted to small clinical trials until recently. In 2017, the US FDA approved two CAR T cell therapies: one for children with the treatment of acute lymphoblastic leukemia (ALL) and one for adults with advanced lymphomas [110]. These are the first batch of approved cancer immunotherapies using fusion proteins. The CAR strategy is a personalized therapy to engineer the patient’s own immune cells to specifically recognize and kill the patient’s cancer cells. Similar to other CARs, the primary concerns of NKG2D CARs therapy should be off-tumor toxicity and cytokine storm. It requires a tricky balance between keeping the CAR T or NK cells active enough to kill tumor cells and controlling them to not be overactive and lead to autoimmune toxicity. Endogenous NKG2D, individual variability, dose control, and combination with other anti-cancer therapy are other issues that need to be taken into account as well.

## Figures and Tables

**Table 1:** Fusion proteins of NKG2D/NKG2DLs in cancer immunotherapy.

Strategy	Fusion Protein	Target/Pathway	Malignancy	References
NKG2DL + Antibody	H60-TNT3	UNA	YAC-1	[35,36]
	Rae1β-TNT3	UNA	CT-26, LLC	[36]
	MICA_E_-Fc (conjugate with Fab)	CEA/HER2/CD20	CC, BC, OC, Raji	[40]
	ULBP2-BB4	CD138	MM	[42]
	anti-HER2 IgG3-Rae1β	HER2	MC	[37]
	ULBP2-anti-PSMA scFv	PSMA	PC	[43]
	MICA_E_-7D8	CD20	CLL, MZL, MCL	[38]
	ULBP2_E_-7D8	CD20	CLL, MZL, MCL	[38]
	ULBP2-anti-CEA	CEA	CC	[44]
	mAb04-MICA_E_	VEGFR	BC	[39]
	anti-VEGFR2 scFv-MICA_E_	VEGFR	HUVEC, K562, MDA-MB-435	[46]
	rG7S-MICA_E_	CD24	HCC	[41]
NKG2DL + Cytokine	MULT1_E_-IL-12	IL-12R	TC-1	[56]
	MICA_E_-IL-12	IL-12R	A549	[57]
	OMCP-mutIL-2	IL-2R	LLC, YAC-1	[58]
NKG2DL + Fas	MULT1_E_-Fas_TI_	Fas	TC-1	[67,68]
NKG2D + Antibody	NKG2D_E_-Fc	ADCC	BC	[69,70]
	Dap10-Fc-NKG2D_E_	–	RMA/RG, P815	[71]
	anti-CD3 scFv-NKG2D_E_	CD3	RMA/RG, P815, ID8, MC-38	[73]
NKG2D + Cytokine	NKG2D_E_-Fc-IL-2	IL-2R	TC-1	[74]
	dsNKG2D_E_-IL-15	IL-15R	CC	[75]
	dsNKG2D_E_-IL-21	IL-21R	CT-26	[80]
NKG2D CARs	NKG2D-CD3ζ_I_	TCR	MM, OC, Lymphoma, BC, etc.	[86,87,88,89,90,91,92,93,94,95,96,97,101]
	NKG2D_E_-CD28-CD3ζ_I_	TCR	ESFT, 4T1.2	[98,101]
	NKG2D_E_-4-1BB-CD3ζ_I_	TCR/CD137	OC	[99]
	DAP10-NKG2D_E_-CD3ζ_I_	TCR	Osteosarcoma, 4T1.2	[100,101]

Subscript formatted E, T and I indicate extracellular domain, transmembrane domain, and intercellular domain, respectively. The NKG2D/NKG2DLs pathway was not included in the Target/Pathway column. UNA, universal nuclear antigen; LLC, Lewis lung carcinoma; CEA, carcinoembryonic antigen; HER2, human epidermal growth factor receptor 2; CC, colon carcinoma; BC, breast carcinoma; OC, ovarian carcinoma; MM, multiple myeloma; MC, mammary carcinoma; PSMA, prostate carcinoma membrane; PC, prostate carcinoma; CLL, chronic lymphocytic leukemia; MZL, marginal zone lymphoma; MCL, mantle cell lymphoma; VEGFR, antigen vascular endothelial growth factor receptor; HUVEC, human umbilical vein endothelial cell; HCC, hepatocellular carcinoma; TCR, T cell receptors; ESFT, Ewing’s sarcoma family of tumors.
